# A quasi-experimental study assessing the effectiveness of a community-based egg intervention in the nutritional and health status of young children from rural Honduras

**DOI:** 10.1371/journal.pone.0312825

**Published:** 2024-11-05

**Authors:** Ana M. Palacios, Mario Keko, Haresh Rochani, Dziyana Nazaruk, Asli Aslan, Joana Tome, Tilicia Mayo-Gamble, Gisela Ramos, Laura Manship

**Affiliations:** 1 Department of Health Policy and Community Health, Jiann Ping Hsu College of Public Health, Georgia Southern University, Savannah, Georgia, United States of America; 2 Department of Biostatistics, Epidemiology and Environmental Health Sciences, Jiann Ping Hsu College of Public Health, Georgia Southern University, Statesboro, Georgia, United States of America; 3 Institute for Water and Health, Georgia Southern University, Savannah, Georgia, United States of America; 4 Hombro a Hombro, Intibucá, Honduras; 5 Shoulder to Shoulder Inc, Dayton, Ohio, United States of America; Arizona State University, UNITED STATES OF AMERICA

## Abstract

**Objective:**

This community-public-private-academic coalition project implemented and evaluated the effectiveness of a rural, community-based egg intervention that aimed to support the nutrition and health of children living in rural, poor communities from Intibucá, Honduras, during the COVID-19 pandemic.

**Design:**

This investigator-blind, non-randomized, controlled study was informed by a community health improvement process and participatory research. Women from 13 communities were given a microloan to start an egg farm that supplied 1 egg daily to 201 children ages 6–24 months for 1 year (intervention group). Control communities (n = 14) were selected from neighboring municipalities with similar sociodemographic backgrounds based on size. Sociodemographic-, anthropometric-, and morbidity data were collected biannually between January 2021 to January 2022. Outcome changes were compared with linear-, generalized- or Poisson- mixed models adjusted by sex, age, maternal education, breastfeeding status, assets, adults living at home, baseline outcomes, and community-cluster.

**Results:**

Baseline to 6- and 12-month weekly frequency of egg intake significantly increased in the intervention vs. the control group: 6-month change = 1.86; 95%CI (1.61, 2.14); 12-month change = 1.63; 95%CI (1.42, 1.87 p<0.001), respectively. Baseline to 12-month changes in the intervention group were not significant for length/height-for-age-z-scores = 0.12, p = 0.187; weight-for-length/height-z-scores = -0.02, p = 0.78; and diarrhea prevalence, AOR = 1.69; 95%CI (0.53, 5.42), p = 0.378. Lower odds of respiratory infections were observed for the intervention vs. the control group at 6- and 12-month post: AOR = 0.28; 95%CI (0.12, 0.63), p = 0.002; AOR = 0.30; 95%CI (0.12, 0.75), p = 0.010, respectively.

**Conclusions and relevance:**

Children in the intervention group reported consuming eggs more days per week relative to the control group. Lower odds of respiratory infections were observed in the intervention group throughout the study. Ongoing follow-up will offer more insights on the intervention’s effectiveness in linear growth, dietary diversity, food security, and other nutritional outcomes.

## Introduction

Malnutrition in Honduras is a widespread public health problem. The most recent national survey of demography and health indicated that 18.7% of children under age 5 are stunted, defined as having a length/height-for-age-z-score (haz) <-2 [1]. In Intibucá, a predominantly rural department located in the Southwestern midlands of Honduras, the nutritional status of children is much worse as about 1 every 3 children under five years old are stunted [1].

In addition to stunting, the region is experiencing increasing rates of overweight and obesity, even in young populations. This increase is disproportionally affecting the lowest socioeconomic quintile of the population, thus highlighting important disparities. For instance, of 10 countries from the region, Honduras exhibits the greatest prevalence increase in overweight and obesity increase among the lowest quintile population, relative to those from higher wealth quintiles [2]. In sum, the double burden of malnutrition is increasing as a response to rapid changes in food systems where high-calorie, non-perishable, low-micronutrient foods are more accessible and cheaper, in addition to other pressures that impact the region related to climate change [2].

Malnutrition in the first few years is associated with greater mortality, and severe morbidity. Later in life, malnutrition is associated with lower learning capacity and impaired development, greater probability of suffering from chronic, non-communicable diseases, and lower economic productivity [3, 4]. Worldwide, the decline in haz occurs primarily during the first two years of life [5]. This period corresponds with the “complementary feeding period” and is thought to be the consequence of poor quality and quantity of complementary foods, [6] in addition to increased infections and inadequate care practices [7].

In Latin America, rural, remote populations, and females systemically suffer from a lack of access to economic opportunities, markets, credits and services, which impacts their ability to generate income, resulting in poverty and food insecurity [8]. Further, rural areas have limited access and availability to healthy, nutritious, and perishable foods, including animal-source foods, which are recommended by the World Health Organization to be offered to young children in the complementary feeding period as frequently as possible [9].

Some evidence in Ecuador showed that providing one egg daily to 6- to 9-month-old children had a significant effect in increasing haz, and in reducing the prevalence of stunting. Further, the study also showed significant positive effects in raising choline pathway blood biomarkers and omega-3 fatty acids, key nutrients for growth and development [10, 11].

A recent systematic review meta-analyzed 9 different interventions including, 3,575 children and observed a statistically significant increase in linear growth, and weight when compared to children in the control groups [12].

Scaling-up evidence-based interventions to improve nutrition and health in low-resource rural settings is challenging. This community-based participatory program aimed to address barriers to access and availability of nutrient-rich eggs to rural, underserved families and young children by modifying the local food systems, in an effort to ameliorate the pressures of access, and availability of nutritious foods, and food insecurity that were exacerbated during the COVID-19 pandemic [13] while promoting local egg production, and female entrepreneurship. The main aim of this study was to assess the effectiveness of the program in the nutritional and health status of 6- to 24-month-old children from 27 rural communities from Intibucá.

## Materials and methods

### Study design and participants

This study followed a quasi-experimental, open-label, investigator-blind, non-randomized community-controlled trial, registered at clinicaltrials.gov with a unique identification number of NCT04721197 in 27 rural communities located in the municipalities of Colomoncagua, Camasca and Concepción (Department of Intibucá, Honduras). IRB approval was provided by the Indiana University Institutional Review Board (IRB), protocol No. 2012965659, the Georgia Southern University IRB No. H22066, and the Asociación Hombro a Hombro 12–2020 ensured the protocol and all procedures were done in accordance with the Honduran General Health Law on Health Research (DOF-02-04-2014) [14]. Written and oral consent were obtained from all parents.

Participant recruitment occurred during the monthly community health program meetings: the AIN-C, (initials in Spanish for *Integral Attention to Childhood in the Community—*Atención Integral a la Niñez en la Comunidad) held in pre-specified community locations [15].

**Fig 1:**
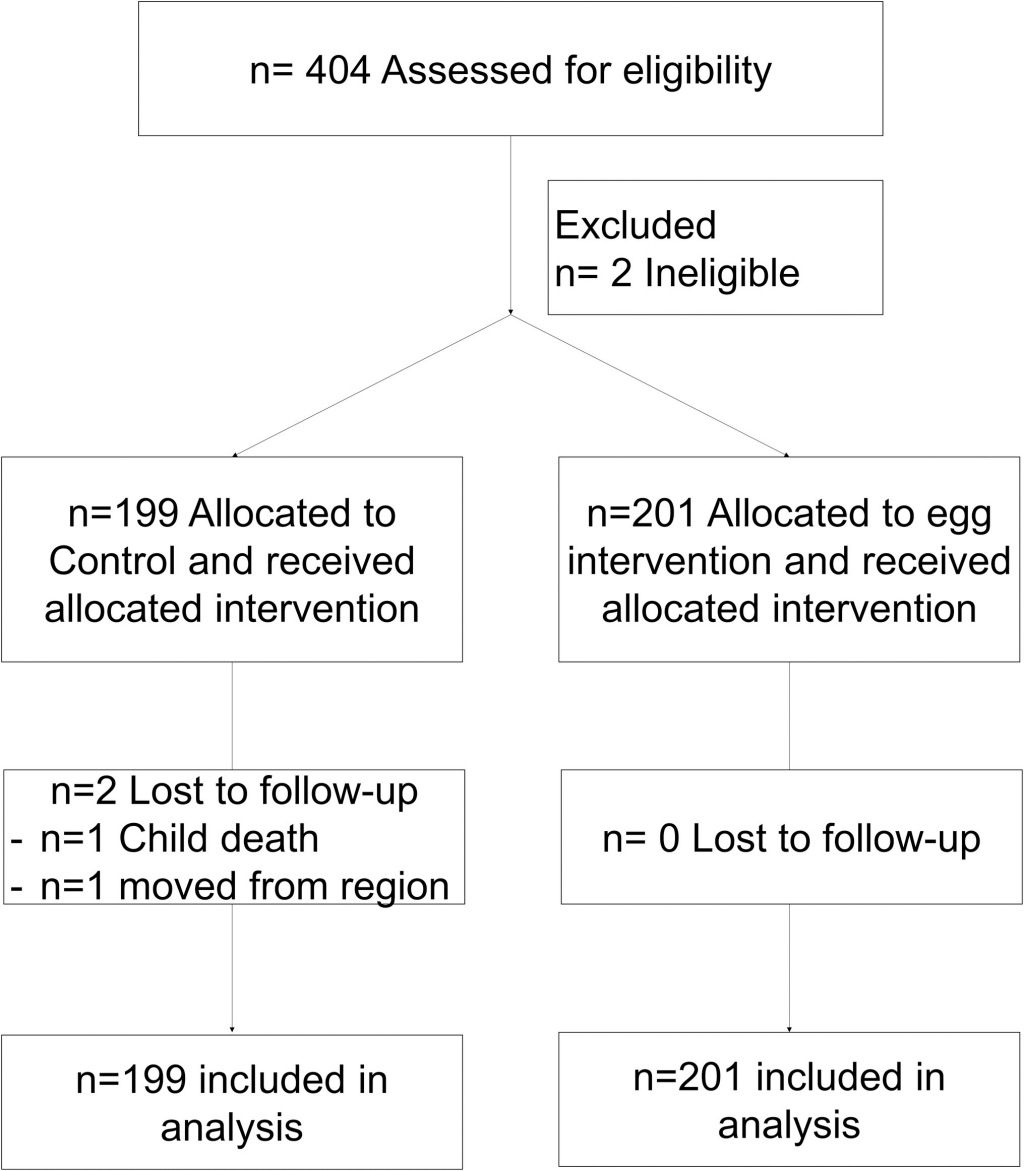
Study consort flow diagram.

Infants and young children ages 6- to 24- months of age at the time the study began (January 2021) were eligible to participate if they lived in the study communities, and did not have a diagnosis of cerebral palsy, birth defect or condition that could affect their growth or development. Children were excluded if they had a known egg allergy, or their families were considering leaving the region within the next 12 months. Details on screening and follow-up are included in Fig 1. Only the co-author Gisela Ramos, field program coordinator, had access to identified data. The rest of the coauthors do not.

### Intervention

The intervention was conceived using a community-health improvement process (CHIP) framework [16] and a socio-ecological model where a coalition was formed to identify, deliver, and monitor this intervention (S1 File).

Participants received the following interventions, depending on the community in which they lived:

- *Egg group*: Mothers of participating infants and toddlers received two vouchers during AIN-C monthly meeting. Each voucher could be exchanged for 15 eggs on a biweekly basis in the local community egg farm, n = 201.
- *Control group*: Mothers of participating infants and toddlers continued to receive the monthly community health visits, and education on breastfeeding and child nutrition, disease prevention and other caregiving practices, n = 199.

### Intervention allocation

The communities that received the egg (n_i = 13) were conveniently selected based on the identification of a motivated female willing to start a community egg farm to provide one egg daily, initially for 12 consecutive months to all children ages 6 to 24 months that at the time the project began, met inclusion criteria, and based on the community’s high need. Each female received a loan sufficient to build a chicken coop in their own parcels, purchase enough hens to provide study-needed eggs, and general chicken care. Egg producers also received basic financial education and a calendar with specific deadlines. A community health worker tracked the monthly farm progress and the loan repayment status.

The intervention communities were purposely matched with similar size and sociodemographic characteristics from neighboring areas to be used as “controls”, n_i = 14. Participants in the control communities received a small gift of $5 dollars or less (e.g., hand or dish soap, baby blanket, cooking pot, a baby blanket or similar) for completing the baseline, 6- and 12-month assessments and continued to receive the AIN-C activities as usual.

### Data collection

After verbal and signed consent, trained community health workers administered a survey to all the participant’s primary caregivers. The survey included family demographics, environmental factors, feeding and caregiving practices [17], a food frequency questionnaire [17], and anthropometry, during the AIN-C community visits at baseline and follow-up timepoints January 2021, July 2021 (6-month), and January of 2022 (12-month post) in both intervention groups.

### Maternal and sociodemographic variables

To determine household economic resources, a questionnaire listing 16 household items and ownership of land and animals was adapted from the 2013 XVI Census (Honduras). An “assets” variable was computed by adding all positive answers indicating ownership or possession of the item. Higher scores indicate more assets in the household [18].

Maternal education was categorized as having completed “secondary or more” education, or “primary or less.”

### Anthropometry

Infants and toddlers’ anthropometry was measured prior to beginning to receive the study eggs by trained community-health workers at baseline, 6- and 12-months post, following Cogill’s 2003 Anthropometric Indicators Measurement Guide [19]. Recumbent length was assessed using a Seca^®^ 417 infant measuring board, Seca^®,^ Chino, CA, for children under 24 months of age, and a locally manufactured measuring board that was calibrated with the Seca infant measuring board prior to each measurement. For older children, standing height was assessed using a calibrated, locally manufactured height board. Infants and children were weighed using the Salter^®^ Mechanical Hanging Scale-25 kg, Salter^®^, Manchester, UK. Weight was measured in kilograms and rounded to the nearest decimal. Anthropometric z-scores were calculated using the 2006 World Health Organization’s Child Growth Standards for children <5 years of age [20].

Stunting was defined as having a haz <-2, underweight as having a weight-for-age z-score (zwaz) <-2, overweight as having a weight-for-length/height z-score (wlz) >2.

### Dietary variables

A semi-quantitative food frequency questionnaire targeted the child’s diet with local common foods during the previous week was adapted using the Latin American and Caribbean Food Insecurity Scale, “ELCSA” for its acronym in Spanish [17].

Weekly animal source foods were computed by adding reported weekly intake of dairy, flesh foods (beef, fish, viscerae, etc.), and eggs (scores ranged from 0 to 18). Greater scores indicated more intake of animal source food intake.

### Child morbidity

Child morbidity was assessed weekly via the caregiver (prior 7-day) recalls for the following morbidities: fever, respiratory symptoms (cough, nasal congestion, soar ear or throat), respiratory infection (cough, nasal congestion, soar ear or throat and fever), and diarrhea (three or more liquid or semisolid stools in 24-hours).

### Process measures

During the trial, we implemented a “strengths, weaknesses, opportunities, and threats (SWOT) [21] analysis in n = 12 mothers and n = 12 egg farm owners to track the progress of the intervention using individual in-depth, semi-structured interviews implemented by a local community worker that was trained to performed this. Interviews were recorded and transcribed by a Spanish speaker individual, and then translated for analysis.

### Primary and secondary outcomes

Primary outcome measures included: changes in haz from baseline at 6-, and 12-months post-intervention. Secondary outcome measures analyzed herein include changes from baseline at 6- and 12-months for the following outcomes: stunting, underweight, diarrhea, respiratory symptoms, and respiratory infections.

### Statistical analysis

The statistical analysis of this study has been previously published here: https://osf.io/4uqc2/. Investigators performing the analyses were blind to treatment allocation. Data were imported to SPSS® statistical software package Version 25.0 (IBM SPSS Inc., Chicago, IL, USA) and checked for validity and data entry errors. Then, data were imported into the SAS (SAS 9.4, Research Triangle Park, NC), where continuous variables were examined for normality. Descriptive statistics were used to characterize the study participants at all time points. An intention-to-treat analysis was performed, which included all participants enrolled at baseline.

For continuous outcomes: haz, zwfl, and zwaz, linear mixed models with repeated measures were used to baseline-to 6- and baseline to 12- month changes. Models included factors for group, time, and group-by-time interaction, using a difference-in-difference approach, and models were adjusted by baseline measures of outcome variables, infant sex, maternal education, household asset scores, infant age, breastfeeding status, and the number of adults at home, as number of adults appears to be associated with better nutritional outcomes in similar contexts in the region [18]. Community-cluster was included as a random effect to avoid an inflation of type I error rates in the results from this study [22]. For the linear mixed models, the assumptions of linearity, homoscedasticity, and normality of the distribution of marginal and conditional studentized residuals were checked graphically (no studentized residuals exceeded l4l; observations that could be outliers were verified if they were “true outliers” (by going back to the data collection files and excluding data entry errors, and by judging if the values were biologically plausible) and were kept in the model.

Baseline to 6- and 12-month changes in weekly egg intake (count of days per week any amount of egg was consumed) were calculated using Poisson generalized linear mixed models, adjusting by age, sex, and maternal education and a random intercept for community cluster.

For binary outcomes assessing the proportion of children with stunting, wasting, and morbidity), generalized linear mixed models were used and were adjusted for sex, age at baseline, maternal education, breastfeeding status, asset score, and number of adults at home. We included time (6 or 12 months), and interaction of time x group as covariates. Marginal odds ratios (OR) for comparing the intervention vs. control groups (reference category) were generated for each timepoint. No deviations from the original protocol were documented.

### Sample size and power analysis

With 200 children expected to be enrolled in the intervention group and 200 in the control, we expect a total of 140 per group to complete 12 months. We would then have 80% power to detect a significant difference (two-sided alpha = 0.05) in outcomes at 12 months (changes from baseline) between groups if the true effect size is at least 0.34 (“med/small”) for Cohen’s d (M1-M2/SD) (where M1 is the mean of the intervention group, M2 is the mean of the control group, and SD is the pooled standard deviation for the two groups), in a simple comparison (t-test) of means if all observations are independent. In actual analysis, all children enrolled with outcome measures from at least one time point will be included in linear mixed models where partial data will be included for those who drop out which will provide higher power than the completers-only analysis. Because children’s data are clustered within each community in mixed models, power is influenced by the correlation of children’s outcomes within communities. In cluster sampling, the power/sample sizes are influenced by the design effect (DE) and interclass correlation from correlation (ICC) within cluster (DE = 1 + (m-1) x ICC, where m is sample size within cluster. With an average of approximately 15 children per community, and a conservative ICC of 0.1, the DE = 1+15*.1 = 2.5. The true sample size of 200 per group may then only be as effective as a sample size of 80 (= 200/2.5), and effect sizes will need to be “medium” d = 0.45 to maintain 80% power with 0.05 type I error rate, with this clustered design.

## Results

Detailed information on screening and follow-up procedures is presented in Fig 1. A total of 404 participants were assessed for eligibility and two children did not meet inclusion criteria. Among these, 201 children living in 13 communities were assigned to receive the intervention, and 199 children living in 14 communities were assigned to the control group. Losses to follow-up were lower than expected (0.5% attrition rate). In the control group, one child died due to complications of an intestinal worm infection, and another one moved out of the study area. Across all the models considered, the presence of missing data was less than 5%. No study-related harms were reported by parents or study staff. S2 File includes the list of study communities, and proportion of children by intervention group.

At baseline, children were 15.53 months-old (SD = 5.57), 50% female. The 74.8% of mothers reported having achieved less than secondary education. At baseline, overall mean haz was -1.09 (SD = 1.19), and mean wlz was 0.39 (SD = 1.12). Table 1 summarizes the sociodemographic and baseline characteristics of the study sample by treatment group.

**Table 1:** Sociodemographic and baseline child characteristics^a^.

Variable	Control (N = 199)	Intervention (N = 201)	P-Value^b^
Age, months	15.46 (5.49)	15.61 (5.66)	0.783
Sex, female (%)	99 (49.75)	101 (50.25)	0.920
Maternal education, secondary or more (%)	54 (27.14)	47 (23.38)	0.388
Household asset score	7.03 (1.78)	4.62 (1.94)	**<0.001**
Currently breastfeeding (%)	156 (78.79)	154 (76.62)	0.603
Egg intake at baseline, days per week	3.20 (1.29)	3.43 (2.11)	0.179
Living at home			
Adults	2.97 (1.39)	3.02 (1.55)	0.733
Children	2.60 (1.36)	2.87 (1.70)	0.081
Length-for-age-z-score	-1.13 (1.12)	-1.06 (1.26)	0.549
Weight-for-length z-score	0.44 (1.09)	0.34 (1.15)	0.387
Weight-for-age z-score	-0.26 (1.03)	-0.29 (1.03)	0.778
Stunting, %	35 (17.59)	38 (18.91)	0.733
Wasting, %	1 (0.51)	2 (1.0)	0.571
Underweight, %	9 (4.55)	8 (3.98)	0.780
Overweight, %	11 (5.56)	14 (6.97)	0.561
Diarrhea, %	21 (10.66)	38 (18.91)	**0.021**
Respiratory symptoms, %	53 (26.63)	43 (21.39)	0.220
Respiratory infections, %	17 (8.54)	7 (3.48)	**0.033**

^a^ Results are means (standard deviations) unless otherwise indicated.

^b^ Comparisons done with Two-tailed independent T-tests for continuous variables or Chi-squares for proportions.

No significant differences were observed between the groups for age, sex, and maternal education at baseline.

At baseline, 17.3% of participants reported not consuming any food from animal sources. This percentage decreased to 9.1% at the midpoint of the study and to 5.0% at the end of the study.

The treatment group had a significantly higher frequency of diarrhea compared to the control group, with 18.91% and 10.66%, respectively (p = 0.021). On the other hand, children in the treatment group had significantly fewer respiratory infections compared to the control group, with 3.48% and 8.54%, respectively (p = 0.033).

At baseline, children in the intervention group reported to eat eggs in average (SD) for 3.43 (2.11) days/week, and children in the control group for 3.20 (1.30) days/week. At 12 months, children in the intervention group reported eating eggs 6.89 (0.49) days/week, and children in the control group reported in average to consume eggs for 3.91 (1.27) days/week.

Weekly egg intake significantly doubled at 6 and 12 months in the intervention group 2.00; 95% CI (1.83, 2.19), and by 2.01; 95% CI (1.83, 2.20), relative to controls, respectively. No significant changes were observed in the control group from baseline to 6-months, 1.08; 95% CI (0.97, 1.20) in the control group, however at 12 months, a mild significant increase in weekly egg intake was observed in the control group, 1.23 95%CI (1.11, 1.37). The difference in changes of egg intake between intervention and control groups from baseline to 6-months was 1.86; 95% CI (1.61, 2.14), p<0.001; and from baseline to 12 months was 1.63 95% CI (1.42, 1.87) indicating a statistically significant greater egg intake in the intervention group, relative to control.

Table 2 displays the estimated 0- to -6 and 0- to 12 month- changes in nutritional status by group, and Table 3 exhibits the prevalence and adjusted odds ratios (AORs) of the study outcomes.

**Table 2:** Baseline to 6- and 12-month post means and comparison changes in primary and secondary outcomes.

Variable	Time^a^	Control^b^ n = 199	Intervention^b^ n = 201	0- to 6-month Effect^c^	p-value	0- to 12-month Effect^c^	p-value
Length-for-age z-score	6	-0.10 (0.07)	-0.05 (0.06)	-0.05 (0.09)	0.596	0.12 (0.09)	0.190
	12	-0.15* (0.07)	-0.27* (0.07)				
Weight-for-length z-score	6	-0.25* (0.07)	-0.18* (0.07)	-0.07 (0.10)	0.439	-0.04 (0.10)	0.662
	12	-0.29* (0.07)	-0.25* (0.07)				
Weight-for-age z-score	6	-0.21* (0.04)	-0.15* (0.04)	-0.06 (0.06)	0.214	0.05 (0.06)	0.411
	12	-0.30* (0.04)	-0.35* (0.04)				

^a^ Time in months

^b^ For each variable, the mean rate of change is adjusted for its measurement at baseline, age at baseline, sex, maternal education, household asset score, number of adults in the household, and breastfeeding at baseline

^c^ Data are presented as adjusted estimated marginal means (standard errors) from linear mixed models (control at 6- or 12-months minus outcome at baseline)–(control at 6- or 12-months minus outcome at baseline)

*Baseline to endpoint change is significant, p<0.05

**Table 3:** Baseline to 6- and 12-month post prevalence changes.

Variable	Time	% Control n = 199	% Intervention n = 201	0-month AOR (95% CI)	p-value	6-month AOR (95%CI)	p-value	12-month AOR (95%CI)	p-value
Stunting	0	35 (17.59)	38 (18.91)	0.81; (0.43,1.55)	0.526	0.88; (0.52,1.49)	0.634	0.96; (0.51,1.78)	0.886
	6	36 (18.37)	42 (20.90)						
	12	39 (20.00)	49 (24.38)						
Underweight	0	9 (4.55)	8 (3.98)	0.38; (0.11,1.3)	0.122	0.64; (0.27,1.52)	0.308	1.08; (0.39,2.99)	0.885
	6	8 (4.08)	7 (3.48)						
	12	11 (5.64)	15 (7.46)						
Diarrhea	0	21 (10.66)	38 (18.91)	1.84; (0.78, 4.35)	0.167	1.76; (0.77, 4.04)	0.181	1.69; (0.53, 5.42)	0.378
	6	12 (6.09)	20 (10.00)						
	12	4 (2.09)	8 (4.02)						
Respiratory symptoms	0	53 (26.63)	43 (21.39)	**0.60; (0.35,1.04)**	**0.07**	**0.58; (0.38,0.90)**	**0.014**	**0.56; (0.34,0.94)**	**0.003**
	6	78 (39.20)	41 (20.40)						
	12	89 (44.72)	68 (33.83)						
Respiratory infections	0	17 (8.54)	7 (3.48)	**0.26; (0.09,0.75)**	**0.012**	**0.28; (0.12,0.63)**	**0.002**	**0.30; (0.12,0.75)**	**0.010**
	6	27 (13.57)	12 (5.97)						
	12	31 (15.58)	15 (7.46)						

^a^ AOR = adjusted odds ratios using the control as the reference “0” category. So, it reads: “there is 70% lower odds of suffering from respiratory infections for the intervention group, relative to the control group at 12 months

In synthesis, no significant baseline to 6- and baseline to 12-month changes in haz, zwfl, zwaz, stunting, wasting, and diarrhea were observed.

The odds of respiratory infections in the intervention group were 72% lower in the intervention group, relative to control at 6-months, AOR = 0.28; 95% CI (0.12, 0.63), and 70% lower at 12-months, AOR = 0.30; 95%CI (0.12, 0.75).

A key finding from the qualitative analysis indicated that families are sharing the egg that should be specific for the participant child, with the whole family.

## Discussion

This community-based participatory research study was co-developed by communities, non-profit organizations, the local health authorities, and the principal investigator’s academic team. The vision was to adopt and scale an egg program to address undernutrition in the region using evidence from a randomized controlled trial implemented in the Ecuadorian highlands, where children ages 6 to 9 months (n = 163) reported a significant increase in length-for-age z score of 0.63; 95% CI (0.38, 0.88) and a 47% decrease in the prevalence of stunting [10].

Although weekly egg intake was two times significantly greater at 6- and 12-months in the intervention versus control group, no associations were observed between the intervention effect and children’s nutritional status, including linear growth, the primary study outcome. We did observe a lower AOR for respiratory symptoms and infections that was sustained throughout the 12 months in children living in the intervention communities, relative to controls.

It is well-documented that undernutrition is associated with increased morbidity and mortality [23]. Chicken eggs are an important source of micronutrients needed for human development and sustenance. Eggs are one of the cheapest animal food sources and are nutritious sources of vitamin A, iron, vitamin B12, riboflavin, choline, zinc, calcium, proteins, and fatty acids [24, 25]. Eggs can be locally sourced and do not immediately need refrigeration, which is a major limitation with other animal-source foods. Eggs also contain other key components involved in immunity [10]. Macrophages are important in both innate and acquired immunity in the defense against virus, bacteria, mycobacteria and fungi in the respiratory track [26], and egg proteins have been associated with multiple immune response pathways, especially modulating macrophage physiology. Ovalbumin has been reported to increase tumoral necrosis factor alpha macrophage secretion [27] and enhance the cell’s phagocytic activity [28]. In animal models, ovotransferrin has been observed to stimulate the promotion of proinflammatory cytokines and metalloproteinases [29], and ovomucine seems to stimulate macrophages through increased synthesis of interleukin-1 and hydrogen [30]. Egg yolk livetins appear to modulate macrophage- proinflammatory cytokines, such as IL-1β, 6, 10, and TNF-α [31]. Through these mechanisms, eggs could support an improved response to infection and lower the incidence of morbidity by common microorganisms.

The lack of observed effectiveness in linear growth could be explained by multiple reasons. First, during our process measures (unpublished data), some parents reported mixing the program-specific eggs with the whole family’s meal, potentially diluting the intervention effect. Many households receive coliform-contaminated water (formative research), and parents and health promoters report frequent diarrhea outbreaks and intestinal parasitism. Third, linear growth has a highly complex and intricate biology [32, 33]. Frongillo et al., 2019 argue that nutrition interventions may benefit children but may not discernibly affect linear growth deficits in immediate or intermediate periods, thus, 12-months may not have been sufficient to observe a statistically significant effect in changes in haz [34].

Our findings are consistent with a trial in rural Malawi which reported that eggs had no impact on growth in young children [35], potentially due to widespread animal source food availability, and better baseline linear growth outcomes. In the present study, we observed that a high proportion of children (82.7%) reported having consumed animal-sourced the prior day, and presented a higher mean haz at baseline (haz = -1.09), higher than in the Ecuador trial, (haz = -1.90). In our study, stunting prevalence was about half of what was reported in the Ecuador trial (18.3% vs. 38.0%), which may be important in our ability to observe the effect of the intervention.

In contrast, a cluster-randomized controlled trial in Burkina Faso provided chickens to families and a behavior change package in n = 250 children ages 4 to 17 months. Children were followed for 10 months, and researchers reported a significant reduction in wasting (β = 0.58; P = 0.03) and underweight (β = 0.47; P = 0.02) [36]. Although our study included some basic behavior-change messaging, we observed opportunities where the messaging and intervention delivery could be improved. Some examples include: 1) offering more recipes with local ingredients, 2) allowing families to pick up the eggs more frequently, and 3) providing more eggs to the families are strategies that we are adopting and currently evaluating.

In sum, we did not observe evidence of the effectiveness of the intervention in linear growth after 12 months, however the program may still have a positive impact on participants’ diet, health, in the families and communities in the longer-term.

This study has multiple strengths including the involvement of members of the community during all aspects of the study, which not only facilitates access to healthy, nutrient-rich food, but also facilitating job opportunities for local rural women. Another strength is the use of implementation science, including process measures throughout the delivery and evaluation of the intervention, which has helped with feasibility and acceptability by the community.

This study has some limitations. First, the study is not randomized, and the intervention communities were selected based on their needs and vulnerability. Through formative research, we tried selecting communities with similar sociodemographic backgrounds, however baseline differences in assets suggest that the control communities may be slightly wealthier. To which extent these baseline differences are meaningful is also questioned as all communities are rural, and with widespread poverty. However, all models were adjusted by household asset score and maternal education to account for these differences. Another limitation is that patterns of egg intake were reported but not actually observed and we only collected frequency (not quantity) on participant’s egg intake.

Anthropometric information was collected via trained community-health workers months but standardized by the program coordinator and local healthcare providers, but the investigators that collected the measurements in the intervention communities may have differed from those who collected data in some of the control communities. Additionally, limitations to generalizability may exist. The study was implemented during the COVID-19 pandemic where access to healthy foods and nutrition supplements was a major challenge.

The evaluation of the second year is ongoing, and modifications based on these findings have been implemented. For instance, we have increased the number of eggs provided for each household to address the sharing issue, and we have increased the age at which children can be eligible to participate to 3 years-old. We also have suggested to exchange vouchers for eggs on a weekly basis, and we have been doing preliminary work to support the community with the water issue. We hope that 18- and 24-month-follow up will offer more insights on the longer-term effectiveness of this community-based egg program that would lead to an effective intervention. This study provided important important real-life implementation implications that will help improve intervention delivery, not only for this study, but for other real-life implementation programs aiming to modify food systems to improve health in communities.

## Supporting information

S1 File Community-health improvement process. (DOCX)

S2 File List of study communities and number, and proportion of children. (DOCX)

S3 File Consort checklist. (DOCX)

S4 File Inclusivity in global research. (DOCX)

S5 File Original protocol. (DOCX)
