# Supplementary material for: A quasi-experimental study assessing the effectiveness of a community-based egg intervention in the nutritional and health status of young children from rural Honduras
Source: PLoS One. 2024 Nov 5;19(11):e0312825. doi: 10.1371/journal.pone.0312825 (PMC11537388; doi:10.1371/journal.pone.0312825)
Supplement: S1 File — (DOCX) [file pone.0312825.s001.docx]

S1 File

Community-health improvement process

Actors:

- Non-Governmental organizations: A non-governmental organization (NGO), Shoulder to Shoulder, Inc. from Dayton, OH and their Honduran extension Hombro a Hombro, Intibuca, Honduras, have helped develop the health infrastructure and local health delivery capacity of several municipalities in the department of Intibucá, Honduras, since 1998. These NGOs have supported the Honduras Ministry of Health’s community-health program AIN-C^16^ and implemented multiple nutrition projects, mostly through the provision of a micronutrient-fortified food, in addition to building clinics and donating medical and dentistry equipment. Political transitions and the COVID pandemic impeded the continuation of the aforementioned nutrition program, which led to the creation of the coalition, who after careful consideration, decided to increase availability and access of eggs to children 6 to 24 months of age, as eggs could be inexpensively produced locally, eggs are well known and widely accepted, and because there is existing evidence that eggs can improve the nutritional status of young children.
- Local health authorities: A strong partnership between the NGOs and local health

authorities are in existence. Community-health workers are aware of the nutrition needs in their communities and are supportive and eager to assist in programs aiming to improve nutrition and health.

- Local heads of municipal government: The Office of the Mayors are supportive of programs that aim to improve the nutrition and health of the communities, and often offer in-kind support such as help with transportation, storage, facilitation of office space and training.
- Families are stressed and aware of the challenges in access to foods and availability of healthy foods, especially for their young children. They

In brief, the framework helped map and identify the following:

Problem: The coalition acknowledged during an informal meeting that there is 1) a lack of economic opportunities in the region; 2) access, availability and affordability of healthy foods is challenging; 3) food insecurity was exacerbated by the COVID-19 pandemic; and 4) most people have monotonous diets based in corn, beans, and pasta.

Priority: To improve the nutrition and health of children under 5 years of age, however, given the potential budget constraints, the priority population should be children under 24 months of age.

Strengths: Among the strengths the coalition identified included 1) great community awareness on the importance of nutrition in health and development, especially of young children; 2) there is a drive to increase economic opportunities in the region and an entrepreneurship spirit among some locals; 3) there is existing infrastructure to deliver nutrition and health interventions; 4) there is local capacity that can be leveraged to lead, coordinate, monitor, train, and disseminate the intervention.

Resources: Existent health centers staffed with health workers, strong ties between the community and families, the local health system, the NGOs, political stakeholders, and academic counterparts, women wanting to generate income, and a local, competent, community health worker who is seen as a leader in several communities and has had experience delivering nutrition/health interventions for a long time and is an excellent manager.
