# Supplementary material for: A quasi-experimental study assessing the effectiveness of a community-based egg intervention in the nutritional and health status of young children from rural Honduras
Source: PLoS One. 2024 Nov 5;19(11):e0312825. doi: 10.1371/journal.pone.0312825 (PMC11537388; doi:10.1371/journal.pone.0312825)
Supplement: S2 File — (DOCX) [file pone.0312825.s002.docx]

S2 File

List of study communities and number, and proportion of children^1^

| Community | Frequency | Percent |
| --- | --- | --- |
| 701 | 15 | 3.8 |
| 702 | 12 | 3.0 |
| 703 | 9 | 2.3 |
| 704 | 13 | 3.3 |
| 705 | 14 | 3.5 |
| 706 | 19 | 4.8 |
| 707 | 14 | 3.5 |
| 708 | 24 | 6.0 |
| 709 | 10 | 2.5 |
| 710 | 19 | 4.8 |
| 711 | 21 | 5.3 |
| 712 | 19 | 4.8 |
| 713 | 12 | 3.0 |
| 801 | 26 | 6.5 |
| 802 | 13 | 3.3 |
| 803 | 14 | 3.5 |
| 804 | 8 | 2.0 |
| 805 | 14 | 3.5 |
| 806 | 7 | 1.8 |
| 807 | 22 | 5.5 |
| 808 | 8 | 2.0 |
| 809 | 9 | 2.3 |
| 810 | 11 | 2.8 |
| 811 | 17 | 4.3 |
| 812 | 25 | 6.3 |
| 813 | 16 | 4.0 |
| 814 | 9 | 2.3 |

^1^ Communities that begin with 7 are the communities that received the egg intervention. Communities that begin with an 8 are the control communities.
