# Supplementary material for: A quasi-experimental study assessing the effectiveness of a community-based egg intervention in the nutritional and health status of young children from rural Honduras
Source: PLoS One. 2024 Nov 5;19(11):e0312825. doi: 10.1371/journal.pone.0312825 (PMC11537388; doi:10.1371/journal.pone.0312825)
Supplement: S4 File — (DOCX) [file pone.0312825.s004.docx]

Inclusivity in global research

PLOS’ policy on inclusivity in global research aims to improve transparency in the reporting of research performed outside of researchers’ own country or community and ensures that PLOS publications reporting global research adhere to high standards for research ethics and authorship. Authors of relevant research articles may be asked to complete the questionnaire below, which outlines ethical, cultural, and scientific considerations specific to inclusivity in global research. This questionnaire may be requested when researchers have travelled to a different country to conduct research, if research uses samples collected in another country, research with Indigenous populations or their lands, or if research is on cultural artefacts. Researchers travelling to another country solely to use laboratory equipment will not normally be required to complete the questionnaire. However, the questionnaire can be requested at the journal’s discretion for any submission – if you have been requested to complete this questionnaire by the PLOS journal you submitted to, please do so.

Please complete the questionnaire below and include this as a Supporting Information file with your manuscript. Note that if your paper is accepted for publication, this checklist will be published with your article in the supporting information files. Please ensure that you reference the checklist in the main body of your manuscript. We suggest adding a subsection ‘Inclusivity in global research’ to your Methods section and adding the following sentence: “Additional information regarding the ethical, cultural, and scientific considerations specific to inclusivity in global research is included in the Supporting Information (SX Checklist)”

The questions have been designed to be applicable to a wide range of study types, and there are subsections for both human subjects research and non-human subjects research. If any of the questions are not relevant to your research please mark them as “N/A” as appropriate.

**Ethical considerations, permits and authorship**

*This section is applicable to all research types.*

Provide details as to who granted permissions and/or consent for the study to take place in the Methods section of your manuscript. This should include the names of **all** ethics boards, governmental organizations, community leaders or other bodies that provided approval for the study. If individuals provided approval refer to these people by their role or title but do not list their name(s).

Reported on page number: 5

If there were any deviations from the study protocol after approval was obtained please provide details of these changes in the Methods section of your manuscript.
Did this study involve local collaborators that are residents of the country where the research was conducted or members of the community studied? If you do not have any authors from said communities, please provide an explanation for this below.

Reported on page number: no deviations from the study protocol have occurred.

*Yes. This is community engaged research that followed Community Health Improvement Process as described in the methods section and the S1 file. Gisela Ramos is a local community leader that played an essencial role in the design, implementation and evaluation of the study, she is the local Principal Investigator and is coauthopring the manuscript.*

Everyone listed as an author should meet PLOS’ criteria for authorship and all individuals who meet these criteria should be included in the author byline, rather than the acknowledgements. For further information please see the journal’s Authorship Policy.

**Human subjects research (e.g. health research, medical research, cross-cultural psychology)**

Did you obtain written informed consent from a representative of the local community or region before the research took place? How did you establish who speaks for the community? Details of written informed consent obtained from study participants should be reported separately in the Methods section of your manuscript.

*This project was conceived by the community through a community health improvement process detailed in the S1 file. The academic investigators were invited to participate in the project by the community (it was the community’s initiative). Briefly, local Heads of Municipal Governments, Families, Community Health Workers a local Non-Profit organization were all involved in deciding/approving all aspects of the project.*

**How did members of the local community provide input on the aims of the research investigation, its methodology, and its anticipated outcome(s)?**

*This has been detailed in the methods section and also in the S1 file:*

*“The intervention was conceived using a community-health improvement process (CHIP) framework [16] and a socio-ecological model ￼ where a coalition was formed to identify, deliver, and monitor this intervention (S1 File).”and in S1 file:*

***Community-health improvement process***

*- Local health authorities: A strong partnership between the NGOs and local health*

*authorities are in existence. Community-health workers are aware of the nutrition needs in their communities and are supportive and eager to assist in programs aiming to improve nutrition and health.*

*- Local heads of municipal government: The Office of the Mayors are supportive of programs that aim to improve the nutrition and health of the communities, and often offer in-kind support such as help with transportation, storage, facilitation of office space and training.*

*- Families are stressed and aware of the challenges in access to foods and availability of healthy foods, especially for their young children. They*

*In brief, the framework helped map and identify the following:*

***Problem****: The coalition acknowledged during an informal meeting that there is 1) a lack of economic opportunities in the region; 2) access, availability and affordability of healthy foods is challenging; 3) food insecurity was exacerbated by the COVID-19 pandemic; and 4) most people have monotonous diets based in corn, beans, and pasta.*

***Priority****: To improve the nutrition and health of children under 5 years of age, however, given the potential budget constraints, the priority population should be children under 24 months of age.*

***Strengths****: Among the strengths the coalition identified included 1) great community awareness on the importance of nutrition in health and development, especially of young children; 2) there is a drive to increase economic opportunities in the region and an entrepreneurship spirit among some locals; 3) there is existing infrastructure to deliver nutrition and health interventions; 4) there is local capacity that can be leveraged to lead, coordinate, monitor, train, and disseminate the intervention.*

***Resources****: Existent health centers staffed with health workers, strong ties between the community and families, the local health system, the NGOs, political stakeholders, and academic counterparts, women wanting to generate income, and a local, competent, community health worker who is seen as a leader in several communities and has had experience delivering nutrition/health interventions for a long time and is an excellent manager.*

- *We held frequent meetings with the stakeholders which provided input throughout the study.*
- *We also developed process mesures during the study involving parents of children and egg farm owners that provided insights to the acceptability and implementation of the project.*

When engaging with the local community, how did you ensure that the informed consent documents and other materials could be understood by local stakeholders?

1. We developed the consent form with the local community health workers. Myself, (the PI and corresponding author of the manuscript is a Native Spanish Speaker, along with three more co-authors which peer-validated the consent. We also asked community health workers to provide feedback on the language, and to socialize the project before beginning, and before data collection they all read the consent outloud, explained all details, and responded to any questions that could have arisen to all participants.

Will the findings of the research be made available in an understandable format to stakeholders in the community where the study was conducted (e.g. via a presentation, summary report, copies of publications, etc.)? Please provide details of how this will be achieved.

- Community meetings have been held and led by local community health workers, where the findings of the evaluation have been shared. We have created and shared multiple presentations with the Stakeholders. Because most of our partners do not speak English, in addition to sharing the publication, we will create a visually agreeable and easy to understand for different audiences in Spanish, so the study staff can show during community health meetings.

**Non-human subjects research using specimens/ animals collected as part of the study, or those housed in archival collections. Examples include archaeology, paleontology, botany and zoology.**

Did the permission you obtained from a local authority to perform the study include an agreement on access to outputs and benefit sharing? This may include procedures to enable fair distribution of the benefits and resources arising from the research performed. Please include any details of Prior Informed Consent and Benefit Sharing Agreements obtained. These may be required by field-specific regulations, for example the Convention on Biological Diversity (CBD) and the associated Nagoya Protocol.

N/A

If the material used in your study was imported, please A) provide the year it was imported and B) indicate whether permits were obtained to import/export the materials used, C) provide details of any permits obtained. If this information is not available, please indicate this.

N/A

If you used archival specimens, please state how the material used in your study was acquired by the institute it is held in and provide details of any permits obtained for the original excavations/ sample collection. If this information is not available, please indicate this.

N/A

How was the potential cultural significance of the materials collected in your study to local communities considered in your research design? Were Indigenous peoples and/or local researchers and institutions involved with archaeological excavations / collection of specimens? If so, please provide a description of their involvement.

N/A

If your manuscript includes photographs of human remains please indicate whether authors obtained permission from descendants or affiliated cultural communities to do so.

N/A
