# Supplementary material for: A quasi-experimental study assessing the effectiveness of a community-based egg intervention in the nutritional and health status of young children from rural Honduras
Source: PLoS One. 2024 Nov 5;19(11):e0312825. doi: 10.1371/journal.pone.0312825 (PMC11537388; doi:10.1371/journal.pone.0312825)
Supplement: S5 File — (DOCX) [file pone.0312825.s005.docx]

# Hens Hatching Hope

## Ana M. Palacios, MD, PhD

**Support Provided by:**

Shoulder to Shoulder, Inc

**Table of Contents:**

**Study Schema**

1. **Background & Rationale**
2. **Objective(s)**
   1. **Primary Objective**
   2. **Secondary Objective**
   3. **Tertiary/Exploratory/Correlative Objectives**
3. **Outcome Measures**
   1. **Primary Outcome Measures**
   2. **Secondary Outcome Measures**
   3. **Tertiary/ Exploratory/ Correlative Outcome Measures**
4. **Eligibility Criteria**
   1. **Inclusion Criteria**
   2. **Exclusion Criteria**
5. **Study Design**
6. **Enrollment/Randomization**
7. **Study Procedures**
8. **Study Calendar**
9. **Reportable Events**
10. **Data Safety Monitoring**
11. **Study Withdrawal/Discontinuation**
12. **Statistical Considerations**
13. **Data Management**
14. **Privacy/Confidentiality Issues**
15. **Follow-up and Record Retention**
16. **References**
17. **Appendix**

**Background & Rationale**

Intibucá, Honduras lies in the middle of the internationally recognized high need area called the “Central American Dry Corridor” by the UN FAO. Poverty in this rural, remote region is ubiquitous, with lack of basic services coverage, and of difficult access. Chronic undernutrition and anemia, common symptoms of chronic nutritional deficiencies, are extremely common, especially in young children under age 5. About 1 every three children show stunting, and half of them have anemia, 13% are underweight; and 3% are wasted.

These rates are among the highest in the Western Hemisphere.

Eggs have been shown to be a cost-effective solution to fighting malnutrition in rural areas of developing countries [1, 2]. A study in Ecuador showed a significant improvement in linear growth, and nutritional status after 6 months of receiving daily eggs in the region.

However, the delivery of eggs is a challenge in the region. Shoulder to shoulder and Hombro a Hombro (non-profit organizations who have been delivering health and nutrition interventions in the area since 1998) will provide vouchers to purchase 30 eggs/month to mothers of children between 6-24 months of age in communities located in Intibucá, a department in Honduras. Our role as researchers will be to evaluate the effectiveness of this egg program. The egg program will occur regardless of our involvement.

Our specific role in this project will be to request anthropometric information in Jan-Jul of 2021 and Jan 2022 from the 6 month to 2 year-old children collected as part of the daily activities of Shoulder to Shoulder and Hombro a Hombro. In addition, we will ask mothers to participate in completing a survey that will collect sociodemographic information, morbidity, feeding practices, and food insecurity. This survey will be asked every 6 months (Baseline (Jan)- 6 month (Jul) and 13 months (Jan, 2022).

The subjects will have an opportunity to listen to the informed consent, to read it, and to ask questions about it. They will also have an opportunity to re-read it again as they receive a physical copy. If the mothers/primary caregiver consent to participate in the study, they will verbally consent to participate in the study, and sign a document that will be kept by the NGO in a stored office in the headquarters of the organization in Intibucá, Honduras. If participants do not consent to participate in the study, they will still continue to receive the egg vouchers, and continue to receive all activities offered by the local non-profit organization.

The informed consent was created in Spanish and is attached with a translation in English. The principal investigator translated the consent to English. Ms. Laura Manship reviewed the English and Spanish versions, and Ms. Gisela Ramos (community health worker) ensured that the language was appropriate for the cultural and educational contexts of the participants. The local NGO collected the signatures of all participants allowing their children to participate in the study, and they are storing them in a locked cabinet in their office headquarters, as it is law to collect a signed consent form in Honduras for any research project, however the principal investigator is requesting the IRB for a waiver of signature of the consents, as it will increase the possibility for a breach of confidentiality.

Finally, our field staff that will be collecting the data do not speak English, they live in a remote, rural region in Honduras, and have a minimal knowledge of navigating on the internet. The CITI training will not be something they will be able to complete, given their context, education level, and the fact that it is largely in English. However we have experience doing research with them, and we are committed to train and strictly follow the ethical principles of human subject research. We have developed a training presentation on ethical aspects of the study.

**Objective(s)**

- 1. **Primary Objective:**

The primary objective of this research is to evaluate the effectiveness of an egg intervention in linear growth.

- To compare the length/height for age z-scores (LAZ) in 6 – 24-month-old children whose mothers are receiving 30 egg vouchers monthly, compared with same-aged children from similar communities that will not be covered by the egg program. Both groups will continue to receive the standard of care.
  1. **Secondary Objectives:**

1. To compare the weight-for age z-scores (WAZ) and underweight (WAZ <-2) prevalence of 6-24 month-old children in an egg program, compared with same-aged children that will receive the standard of care (controls).
2. To compare the weight-for-length/height (WHZ) z-scores and wasting prevalence (WHZ <-2) of 6-24 month-old children in an egg program, compared with controls.
3. To evaluate the effectiveness of an egg intervention in rapid weight gain (zBMI upward percentile crossing of >+0.67 SD) in 6-24 month-old children in an egg program, compared with controls.
4. To evaluate the effectiveness of the egg intervention in the prevalence of reported diarrhea and respiratory infections in 6-24 month-old children from the egg program, compared with controls.
5. To evaluate the effectiveness of the egg intervention in the prevalence of sick-child consultations and hospitalizations in the health centers, of 6-24 month-old children from the egg program, compared with controls.
6. **Outcome Measures/Endpoints**
   1. Primary Outcome Measures:

- Significantly higher length for age z-scores in children that received the egg intervention vs. controls at 6 and 12 months.
  1. **Secondary Outcome Measures**

A1. A significantly lower prevalence of stunting in children who received the egg intervention vs controls at 6 and 12 months

B2. A significantly lower prevalence of underweight in children who received the egg intervention vs. controls at 6 and 12 months.

D1. A significantly lower prevalence of rapid weight gain in children that received the egg intervention vs. controls at 12 months.

E1. A significantly lower prevalence of diarrhea in children that received the egg intervention vs. controls at 12 months.

D2. A significantly lower prevalence of respiratory infections in children that received the egg intervention vs. controls at 12 months

- Significant reduction in food insecurity in children in the egg program vs. controls
- Significant improvement in dietary diversity in children in the egg program vs. controls
- No observed changes in breastfeeding practices in children from either group

1. **Eligibility Criteria**
   1. **Inclusion Criteria**

- Healthy children ages 6 to 24 months at the time the study begins
- No cerebral palsy, birth defect or condition that may affect their growth or development.
- Children living in Intibucá, Honduras in Camasca, Concepción, Colomoncagua, Santa Lucía, or San Marcos de Sierra
  1. **Exclusion Criteria**
- Children from families that are considering leaving the region within the next 12 months
- Known egg allergy

1. **Study Design**

This is a prospective, community-cluster study that will evaluate the effectiveness of a program that will provide 30-day egg vouchers to mothers of 6 to 24 month-old children living in rural areas of Intibucá, Honduras. The program will be delivered by a local non-profit organization (Shoulder to Shoulder - Hombro a Hombro). This research study consists of a survey that will be implemented at baseline, 6 months and 12 months. (Jan 2021-Jul 2021-Jan 2022)

***Flow diagram***

Prior to

Total N:402 **Obtain informed consent. Screen potential participants by inclusion and exclusion criteria.**

Enrollment

**Perform baseline assessments**

Sociodemographic, food insecurity, dietary, family structure, and morbidity survey prior to beginning the program.

Visit 1

Jan 2021

**Midline assessments:**

Dietary, morbidity, anthropometry, food insecurity survey, adherence

Visit 2

July 2021

**Endline assessment:**

Dietary, morbidity, anthropometry, food insecurity survey, adherence

Visit 3

Jan 2022

1. **Enrollment**

The AIN- C program is a community-based health promotion and prevention program that the Pan American Health Organization and World Health Organization created and was adopted to the Honduran context to bring preventative health and nutrition services to children in remote rural areas in Honduras, in the Department of Intibucá. A non-profit organization has been partnering with the government for the past decade to help deliver health into these remote areas in Honduras.

This NGO has developed a program that seeks to improve the nutritional status of young children, and will provide egg vouchers to women who mother children ages 6 to 24 months in January of 2021.

A total of 402 mothers of children will be invited to participate in the survey. An estimated half of which will be from the communities that are covered by the egg intervention and half of children that will be receiving the usual standard of care that live in other communities that will not be covered by the egg intervention.

The communities that received eggs will be matched with similar size and sociodemographic communities from neighboring municipalities to be used as “controls”.

During the government-NGO usual community-health visit, the field workers will explain the purpose of the study. They will also read the informed consent outloud, in addition to providing everyone a physical copy of the consent. In addition, during confirmation of enrollment, the mother will have the opportunity to read the consent again, and voice any questions they may have on their participation. If the mother agrees to participate, then they will proceed to sign the informed consent and medical records -PHI release forms. After these consents have been signed, then the field researchers will proceed to complete the survey. The same survey will also be completed at 6 months and at 12 months. Adherence surveys will be implemented in the communities that are receiving the egg program bimonthly.

1. **Study Procedures**

Both the intervention and control groups will receive the scheduled, standard attention provided during the community-health sessions by the Honduras health services, plus any additional activities planned by the NGO. Even if they don’t want to participate.

The field staff that works for Shoulder to Shoulder will be implementing the survey. The field staff has experience doing research programs and will receive training in ethical principles of human subject research before the study begins.

The surveys will be shared electronically with the principal investigator (Dr. Palacios). These data will not contain any names, or home addresses. The field staff will transcribe the surveys to a database, however if they fall short, the co-investigator may send photos of the completed surveys via an IU secured shared drive specific for PHI, so that the Principal Investigator can assist transcribing the surveys into a spreadsheet (see survey annex). As you can see, the survey doesn’t have names, addresses, or phone numbers.

At 6 and 12 months, we will ask the participants to complete the survey. Participants receiving the egg intervention only will also complete a small adherence survey every two months.

**In-depth interviews and process evaluation**: In addition to the structured survey, we will perform in-depth interviews and a short semi-structured survey to women who are the egg farm owners, and to mothers who are receiving the intervention. A maximum of 30 women will be interviewed. These interviews will be audio recorded, but no additional information that may identify them will be collected. The objective of these interviews will be to assess the perceptions of the egg study, identify challenges, opportunities and to evaluate the impact of the egg program in other aspects such as income generation, knowledge around chicken rearing, and opportunities to improve the delivery of the intervention.

1. **Study Calendar**

|  | Screening | 6-month visit | 12-month |
| --- | --- | --- | --- |
|  | Baseline  (1 month) | Mid  (1 month) | Endline |
| **STUDY PROCEDURES** |  |  |  |
| Study socialization | x |  |  |
| Consent | x |  |  |
| Survey | x | x | x |
| In-depth interviews |  | x | x |
| Provision of Vouchers to purchase eggs (monthly) | x | x | x |
| Anthropometric measurements | x | x | x |
| Education (monthly) | x | x | x |
| Adherence surveys (monthly) | x | x | x |

1. **Reportable Events**

An adverse event would be a breach in confidentiality. If this is the case, the Principal investigator will immediately notify the IRB, and will proceed to delete any documents that have compromised the participant’s identity.

1. **Data Safety Monitoring**

We will review participant’s data entry upon completion of baseline, midline and endline assessments.

1. **Study Withdrawal/Discontinuation**

The participant can manifest their desire to withdraw from the study at any time. They will just have to notify Gisela Ramos, the field coordinator that they no longer wish to participate and she will immediately notify the principal investigator and remove them from the study. The participant will be able to continue receiving any activities that will be receiving without any changes to that.

1. **Statistical Considerations**

To evaluate the intervention effectiveness on growth outcomes, we will use linear mixed models for continuous variables (after transforming if variables do not exhibit a normal distribution) and generalized linear mixed models with logit link for binary outcomes. Full preanalysis plan is published here: <https://osf.io/3gx4e>.

Participant data will be analyzed following an intention-to-treat principle and all available participant data for the particular outcome at baseline,6 and 12 months will be included in analysis. Models will include fixed effects for group, time, and the group x time interaction; whereas, random effects will be used for each child (for correlation of repeated measures over time) and community (for correlation of children within community), to avoid an inflation of type I error rates in the results. All models will be adjusted by age, sex, and baseline growth outcomes. ​

With 200 children enrolled in the intervention (egg) group and 200 in the controls, we expect 140 per group to complete all 12 months. We would then have 80% power to detect a significant difference (two-sided alpha=0.05) in outcomes at 12 months (changes from baseline) between groups if the true effect size is at least 0.34 (“med/small”) for Cohen’s d (M1-M2/SD), in a simple comparison (t-test) of means if all observations are independent. In actual analysis, all 402 children will be included in linear mixed models where partial data will be included for those who dropout which will provide higher power than the completers-only analysis. Because children’s data are clustered within community in the mixed models, power is influenced by the correlation of children’s outcomes within communities. In cluster sampling, the power/sample sizes are influenced by the design effect (DE) and interclass correlation (ICC) from correlation within cluster (DE = 1 + (m-1) x ICC, where m is sample size within cluster.) With an average of approximately 15 children per community (m), and a conservative ICC of 0.1, the DE=1+15*.1=2.5. The true sample size of 200 per group may then only be as effective as a sample size of 80 (=200/2.5), and effect sizes will need to be “medium” d=0.45 to maintain 80% power with 0.05 type I error rate, with this clustered design.

**For qualitative information**, direct content analysis to organize and examine the data will be completed by two different researchers. Categories will be identified and coded to determine consistency, frequency, and context until saturation. Information will be then synthesized and analyzed.

1. **Statistical Data Management**

The field researchers will collect data in paper surveys. The paper surveys will not be identified with the name of the participant but with a code that will be linked to a list with the names and codes of the participants and will be stored in a locked cabinet. The paper surveys will also be stored in a lock cabinet in a secured office in the headquarters of Shoulder to Shoulder/Hombro a Hombro until the study finalizes. The field staff will transcribe those surveys to an excel spreadsheet. In the case that they are falling behind, they will take photos of the survey answers. The photos will be uploaded to an IU secured shared drive in either google drive or Microsoft teams so that the IU-investigator can help digitalizing the answers into the excel spreadsheet.

The field researchers will collect the medical records from the health centers database of only the children who agreed to participate in this study. They will remove any personal identifiers from this medical record database, and include the unique number assigned to the child. This de-identified database will also be uploaded in an IU secured drive to be shared with the PI.

Once these photos have been digitalized, they will be eliminated immediately. The database with identifiers will be stored in an IU secured drive. All data will be scrutinized for plausibility and data entry errors.

1. **Privacy/Confidentiality Issues**

To protect the participant’s privacy and confidentiality, the field staff will collect data in a private space. The surveys collected will not have names, home addresses or phone numbers. Only a code that can only be linked by the field researchers if needed. The data shared with the US investigators of the 402 children will have the child’s sex, date of birth, and municipality (after unblinding). The US team will not have any direct contact with any participant, and the field staff will only contact the participants during the monthly community-health activities where is expected to connect with the researchers, unless participants actively reach out to the field staff.

We cannot guarantee absolute confidentiality. The participants personal information may be disclosed if required by law. No information which could identify the participants will be shared in publications about this study.

Organizations that may inspect and/or copy your research records for quality assurance and data analysis include groups such as the study investigator and his/her research associates, and collaborators, the Indiana University Institutional Review Board or its designees, the Indiana Center for Translational Research (CTSI), and any state or federal agencies who may need to access your medical and/or research records (as allowed by law).

1. **Follow-up and Record Retention**

Deidentified records will be maintained indefinitely. Identifiers except for child’s sex will be erased about 24 months after the data collection aspect and publications of the study have been completed.

1. **References**

1. Iannotti, L.L., et al., *Eggs early in complementary feeding increase choline pathway biomarkers and DHA: a randomized controlled trial in Ecuador.* Am J Clin Nutr, 2017. **106**(6): p. 1482-1489.

2. Iannotti, L.L., et al., *Eggs in Early Complementary Feeding and Child Growth: A Randomized Controlled Trial.* Pediatrics, 2017. **140**(1).
